# The Association of the Essential Amino Acids Lysine, Methionine, and Threonine with Clinical Outcomes in Patients at Nutritional Risk: Secondary Analysis of a Randomized Clinical Trial

**DOI:** 10.3390/nu16162608

**Published:** 2024-08-08

**Authors:** Carla Wunderle, Luana Haller, Rahel Laager, Luca Bernasconi, Peter Neyer, Franziska Stumpf, Pascal Tribolet, Zeno Stanga, Beat Mueller, Philipp Schuetz

**Affiliations:** 1 University Department of Medicine, Internal and Emergency Medicine, Cantonal Hospital Aarau, 5001 Aarau, Switzerland; carla.wunderle@ksa.ch (C.W.); franziska.stumpf@geskes.ch (F.S.);; 2 Department of Health Sciences and Technology, ETH Zurich, 8092 Zurich, Switzerland; 3 Medical Faculty, University of Basel, 4056 Basel, Switzerland; 4 University Hospital of Child and Adolescent Psychiatry and Psychotherapy, University of Bern, 3010 Bern, Switzerland; 5 Institute of Laboratory Medicine, Cantonal Hospital Aarau, 5001 Aarau, Switzerland; 6 Department of Health Professions, Bern University of Applied Sciences, 3008 Bern, Switzerland; 7 Vienna Doctoral School of Pharmaceutical, Nutritional and Sport Sciences, University of Vienna, 1030 Vienna, Austria; 8 Department of Diabetology, Endocrinology, Nutritional Medicine, and Metabolism, University Hospital and University of Bern, 3010 Bern, Switzerland; 9 Faculty of Biomedical Sciences, Università della Svizzera Italiana (USI), 6900 Lugano, Switzerland

**Keywords:** mortality, sarcopenia, malnutrition, biomarker, nutritional support, muscle health

## Abstract

Lysine, methionine, and threonine are essential amino acids with vital functions for muscle and connective tissue health, metabolic balance, and the immune system. During illness, the demand for these amino acids typically increases, which puts patients at risk for deficiencies with harmful clinical consequences. In a secondary analysis of the Effect of Early Nutritional Support on Frailty, Functional Outcomes, and Recovery of Malnourished Medical Inpatients Trial (EFFORT), which compared individualized nutritional support to usual care nutrition in patients at nutritional risk, we investigated the prognostic impact of the lysine, methionine, and threonine metabolism. We had complete clinical and amino acid data in 237 patients, 58 of whom reached the primary endpoint of death at 30 days. In a model adjusted for comorbidities, sex, nutritional risk, and trial intervention, low plasma methionine levels were associated with 30-day mortality (adjusted HR 1.98 [95% CI 1.16 to 3.36], *p* = 0.01) and with a decline in functional status (adjusted OR 2.06 [95% CI 1.06 to 4.01], *p* = 0.03). The results for lysine and threonine did not show statistically significant differences regarding clinical outcomes. These findings suggest that low levels of methionine may be critical during hospitalization among patients at nutritional risk. Further studies should investigate the effect of supplementation of methionine in this patient group to improve outcomes.

## 1. Introduction

Disease-related malnutrition (DRM) impacts as many as 30% of all hospitalized medical patients and over 45% of hospitalized elderly patients [1,2,3]. DRM has been shown to increase the risk of longer hospital stays, reduced quality of life, sarcopenia, infections, and mortality in elderly and polymorbid patients [4,5,6,7,8]. The Effect of Early Nutritional Support on Frailty, Functional Outcomes, and Recovery of Malnourished Medical Inpatients Trial (EFFORT) is, next to smaller trials, the largest study to date demonstrating the beneficial effect of individualized nutritional support in this patient population [6,9,10]. However, while the EFFORT focused on an individualized nutritional strategy to reach individual nutritional targets, certain groups of malnourished patients may benefit from more specific, personalized nutritional interventions [11]. There is a strong interest in studying specific blood biomarkers, including metabolomics and single amino acids, to identify patients for distinct nutritional treatments [12,13,14,15]. Such individualized approaches may further increase the effect of nutritional interventions.

Among potential markers, lysine, methionine, and threonine are three of nine essential amino acids, which are vital components of several metabolic processes such as protein synthesis and the modulation of immune responses [16]. Recent studies have suggested an association of mammalian target of rapamycin (mTOR) signaling and lysine, methionine, and threonine [17,18]. mTOR is a key regulator of cell growth, metabolism, and protein synthesis [19,20]. Essential amino acids, especially methionine, have been shown to be the main activators of mTOR signaling and, therefore, are key players in anabolic mechanisms, promoting muscle protein synthesis and growth [21,22]. Since the human body is unable to synthesize these amino acids, dietary intake is crucial to ensure adequate supply [23]. This may be a limiting factor in chronically ill patients at risk for malnutrition and low food intake. Importantly, during acute illness, the demand for these amino acids typically increases, which puts patients at high risk for deficiencies with negative clinical consequences. Indeed, low levels of lysine, methionine, and threonine were associated with sarcopenia [4] and mortality [10,24] in previous research. An improved understanding of the role of key amino acids like lysine, methionine, and threonine in the pathophysiology of DRM is crucial for developing more effective nutritional interventions [6,7].

Herein, we analyzed the prognostic and predictive significance of lysine, methionine, and threonine regarding clinical outcomes and response to nutritional therapy in patients at nutritional risk included in the EFFORT [6]. By examining the levels of these amino acids and their impact on patient health, this research seeks to provide valuable insights into optimizing nutritional strategies for those suffering from DRM.

## 2. Materials and Methods

### 2.1. Study Design

The EFFORT [6] is a pragmatic, open-label, investigator-initiated, multicenter, randomized controlled trial (RCT). The trial investigated the effects of individualized nutritional support compared to standard-of-care nutrition, namely hospital food, in medical inpatients at nutritional risk on clinical outcomes in eight Swiss hospitals between April 2014 and February 2018. The study protocol was approved by the Ethics Committee of Northwest and Central Switzerland (EKNZ) in January 2014 (registration ID 2014_001) and registered at ClinicalTrials.gov (NCT02517476).

### 2.2. Patient Population

Overall, 2088 patients were enrolled in the EFFORT with the following inclusion criteria: age ≥ 18 years, Nutritional Risk Screening (NRS) total score of ≥3 points, an expected hospital stay of more than 4 days, and willingness to give informed consent within 48h after hospital admission. The NRS is a validated risk screening tool for malnutrition, which assesses the patient’s disease severity and its impairment of nutritional status [25]. Informed consent was provided by all patients or their authorized representatives. Initial admission to intensive care or surgical units was a major exclusion criterion. Additionally, patients were excluded if they were unable to consume food orally; were already receiving nutritional support on admission; or had certain specific medical conditions such as anorexia nervosa, acute pancreatitis, or terminal condition as previously described and published in the main publication [6].

For this analysis, only patients from the Medical University Clinic at the Cantonal Hospital Aarau with available measurements of lysine, methionine, and threonine were included.

### 2.3. Study Intervention (Randomization/Procedures)

Patients were randomized by a computer and assigned to either the standard hospital food group (control group) or the individualized nutritional support group (intervention group). In the control group, patients received standard hospital food without nutritional counseling or additional nutritional support. The intervention group received nutritional support, which was initiated within 48 h after hospital admission and immediately after randomization. Investigators and participants were always aware of their group assignment. However, blinded study nurses conducted the telephone interviews after hospital discharge. Registered trained dietitians provided individual nutrition therapy to reach energy targets calculated with the Harris–Benedict equation [26] for prediction and protein targets. Individualized goals were defined for each patient with a plan based on oral nutrition provided by the hospital or oral nutritional supplements (ONSs). If 75% of the daily energy or protein goals were not reached, enteral or parenteral feeding was recommended within 5 days to avoid further nutritional deficits.

### 2.4. Analysis of Blood Biomarkers

Blood sample collection was carried out by venipuncture following the hospital’s routine processes with preidentified Vacutainer Plasma Separator Tubes (Beckton Dickinson, Allschwil, Switzerland) upon study inclusion. Samples were immediately processed, sent to the laboratory, centrifuged without delay for 5 min at 3000× *g*, frozen in aliquots, and stored at a controlled temperature of −80 °C until further analysis. No freeze–thaw cycles were performed on the aliquots intended for metabolomics measurements. Admission plasma metabolites were analyzed from February to April 2019 by liquid chromatography coupled with tandem mass spectrometry (LC-MS/MS). An Ultimate 3000 UHPLC (Thermos Fisher, San Jose, CA, USA) system coupled to a Sciex QTRAP 5500 mass spectrometer (Sciex, Darmstadt, Germany) was used for targeted metabolomics analyses [27,28,29]. The commercially available AbsoluteIDQ^®^ p180 kit (BIOCRATES Life Sciences AG, Innsbruck, Austria), which showed reliability in an inter-laboratory assessment, was applied for all samples in random sequence [30,31,32]. Undetectable signals for lysine, methionine, and threonine were excluded from the analysis, which resulted in 237 valid measurements of each amino acid.

### 2.5. Outcomes

The primary outcome was defined as 30-day all-cause mortality. Secondary outcomes were mortality at 180 days; the composite endpoint of adverse clinical outcomes within 30 days, which has already been described [6]; a decline in the functional status of ≥10% measured by the Barthel index; the total length of hospital stay; and the incidence of falls during the 180-day follow-up period. The Barthel index ranges from 0 to 100 points, with a higher score indicating better performance with daily activities and quality of life [33]. On days 30 and 180, follow-up interviews were performed with blinding to group assessment. For survival verification, family members or the patient’s family doctor were contacted. For a better assessment of functional outcomes, specifically muscle health and function, the collected routine CT data were added to the existing dataset to analyze the association of lysine, methionine, and threonine on four selected variables: low muscle radiodensity, low skeletal muscle index, high intramuscular adipose tissue, and sarcopenia [34,35].

### 2.6. Definition of Low Muscle Mass and Muscle Radiodensity Based on Abdominal CT Scans

The detailed methodology for assessing muscle mass and composition has been previously documented [34]. CT images of the third vertebra (L3 level) were collected within 3 months of enrollment in the EFFORT and reviewed by research assistants. Radiodensity analysis was conducted on the original DICOM (Digital Imaging and Communications in Medicine) files, which were not contrast-enhanced. For this analysis, four variables were used to define low muscle mass and muscle radiodensity: low muscle radiodensity (MR), low skeletal muscle index (SMI), high intramuscular adipose tissue (IMAT), and sarcopenia.

Mean muscle radiodensity (MR) was categorized according to cut-off values, expressed in Hounsfield units (HUs), proposed by Martin et al. [36]. Measurements of total muscle areas were normalized by height (m^2^) for the SMI [34]. IMAT and HUs are both reliable indicators of muscle composition; therefore, high IMAT was analyzed by calculating quartiles, and they were compared to each other since previous research has found associations with clinical outcomes [37]. The variable for sarcopenia was defined via the combined presence of hand grip strength (HGS) and low MR.

### 2.7. Statistical Analysis

Patients were divided into two groups based on low compared to high lysine, methionine, or threonine levels. The Liu method, which uses the ROC analysis, was used to calculate optimal cut-off values for each amino acid for 30-day mortality [38]. The calculated cut-off value for lysine was 192.5 μmol/L, for methionine 17.45 μmol/L, and for threonine 88.15 μmol/L. We evaluated the normal distribution of all metabolites analyzed by visual assessment of qq plots and histograms. Logistic regression was used to assess associations between binary outcomes and metabolite levels, reported in odds ratios (ORs), and linear regression was used for continuous outcomes, reported in coefficients (Coef.). The same principle of regressions was performed for secondary outcomes. Cox regression models reporting hazard ratios (HRs) were used for mortality outcomes at 30 and 180 days. Analyses were adjusted for randomization group, sex, age, Charlson Comorbidity Index (CCI), and baseline nutritional status (NRS total score) [39]. Kaplan–Meier curves were plotted to visualize data. To compare frequencies of categorical variables, we used Pearson’s chi-squared test, and for continuous variables, a two-sample *t*-test was used. STATA 18.0 was used for statistical analysis. A *p* value of <0.05 and a 95% confidence interval (CI) indicated statistical significance.

## 3. Results

### 3.1. Patient Population

Out of the 2088 enrolled patients, 237 from one center were included in this secondary analysis. Of these 237 patients, 115 were randomized to the intervention group and 122 were in the control group (Supplemental Figure S1). Notably, 122 patients had low lysine levels (cut-off value 192.5 μmol/L), 70 patients had low methionine levels (cut-off value 17.45 μmol/L), and 110 patients had low threonine levels (cut-off value 88.15 μmol/L). The most important admission diagnoses were infection (27.0%), cancer (31.6%), and cardiovascular disease (10.1%). Individual baseline characteristics stratified by the primary endpoint 30-day all-cause mortality are shown in Table 1. Further baseline tables stratified by high and low levels of the individual amino acids can be found in Supplemental Tables S1–S3.

### 3.2. Association of Lysine, Methionine, and Threonine with Nutritional Parameters

In the first step, we investigated the association of lysine, methionine, and threonine levels with nutritional parameters, including the NRS total score and its components BMI, weight loss, and food intake (Table 2). An NRS ≥ 5 points was negatively associated with lysine levels (Coef. −24.4 [95% CI −43.0 to −5.8], *p* = 0.01), suggesting that patients at high risk for malnutrition had 24.4 μmol/L lower mean lysine levels at admission compared to patients with at lower risk (NRS 3 points). Food intake of 50–75% in the week before hospitalization was also associated with lower lysine levels (Coef. −30.2 [95% CI −58.0 to −2.4], *p* = 0.03). No significant association was observed between methionine and threonine and any of the investigated nutritional parameters.

### 3.3. Association of Lysine, Methionine, and Threonine with Mortality and Further Clinical Outcomes

**Figure 1:**
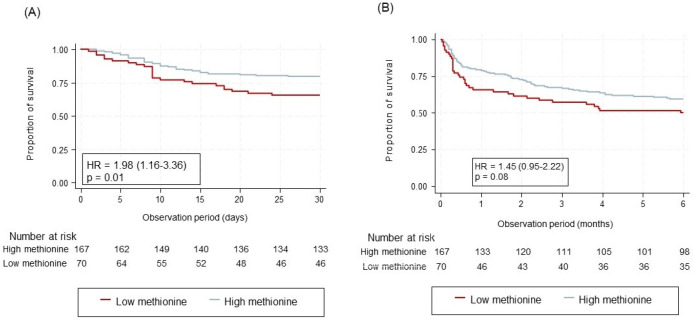
Kaplan–Meier curves for methionine: (**A**) 30-day all-cause mortality, (**B**) 180-day all-cause mortality. Both figures are based on high (>17.45 µmol/L) versus low methionine levels (≤17.45 µmol/L). All hazard ratios shown are adjusted for CCI, sex, NRS total score, and intervention. HR, hazard ratio. CCI, Charlson Comorbidity Index. NRS, Nutritional Risk Screening 2002.

In a model adjusted for comorbidities, sex, nutritional risk, and trial intervention, low plasma methionine levels were associated with 30-day mortality (adjusted HR 1.98 [95% CI 1.16 to 3.36], *p* = 0.01) and with a decline in functional status (adjusted OR 2.06 [95% CI 1.06 to 4.01], *p* = 0.03). Low methionine also showed an association with the risk of adverse events within 30 days (HR 2.21 [95% CI 1.21–4.05], *p* = 0.01). Another finding was a borderline association between threonine and falls within 180 days (HR 2.46 [95% CI 0.98–6.19], *p* = 0.05) (Figure 1). Low lysine, however, showed no association with mortality or secondary clinical outcomes. Results are shown in Table 3.

### 3.4. Association of Lysine, Methionine, and Threonine with Muscle-Specific Outcomes

Among the four muscle-specific outcomes tested, low lysine was significantly associated with sarcopenia (OR 0.37 [95% CI 0.14–0.95], *p* = 0.04). Also, a trend was found between low threonine and low skeletal muscle index (OR 3.14 [95% CI 0.89–11.02], *p* = 0.07) (Table 3). For methionine, no significant associations with muscle-specific outcomes were found.

### 3.5. Association of Lysine, Methionine, and Threonine on Responding to Nutritional Support

**Figure 2:**
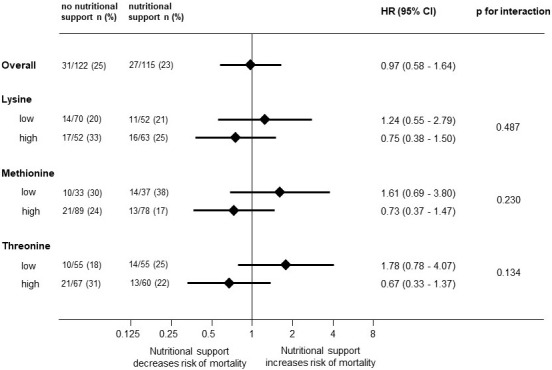
Forest plot for 30-day mortality and subgroup analysis for response to nutritional support. HR, hazard ratio. Adjusted for CCI, sex, NRS total score, and intervention. CCI, Charlson Comorbidity Index; NRS, Nutritional Risk Screening 2002.

For the investigated amino acids, the analysis revealed no significant differences in the response to nutritional support of patients of different subgroups in terms of mortality. While higher levels of the investigated amino acids indicated trends in better treatment response, these were not significant in interaction analysis (Figure 2).

## 4. Discussion

Within this secondary analysis of the randomized EFFORT, we investigated the prognostic potential of three essential amino acids, namely lysine, methionine, and threonine, among medical inpatients at nutritional risk. We observed that low levels of methionine predicted mortality and other adverse clinical outcomes, suggesting that this illness-related amino acid deficiency may negatively affect the clinical course of patients at nutritional risk. Several findings of this analysis have clinical implications and are worth further discussion.

First, when comparing the mean values of the three amino acids from a healthy cohort from France using the same research metabolomics kit as in our cohort, similar levels to our hospital patient cohort were found. In the French healthy cohort, threonine (mean: 127.9 µmol/L) was slightly higher in the French healthy cohort compared to patients in our cohort (mean: 98.3 µmol/L), while lysine (mean: 197.4 µmol/L vs. mean: 197.2 µmol/L) and methionine (mean: 25.4 µmol/L vs. mean: 23.3 µmol/L) were in the same range [30]. This suggests that in patients at nutritional risk, levels of the investigated amino acids remain stable in a majority of patients. However, we found an inverse correlation between a higher risk for malnutrition, as assessed by the NRS total score, and lower lysine levels. This may suggest that patients with low food intake and a corresponding high risk for malnutrition are also at higher risk for deficiencies in this essential amino acid. This observation, however, was not true for methionine or threonine.

Second, low levels of methionine were associated with a two-fold increase in the risk of short-term all-cause mortality and adverse events within 30 days. This means that low methionine at hospital admission was a risk factor, and these patients had a significantly increased risk of dying or having a poor outcome within a short period of time. In line with this finding, a previous study has found low methionine levels to be prognostic in patients with sepsis and predictive of clinical outcomes [40]. Specifically, this study found that baseline methionine levels were significantly lower in patients with more severe sepsis and lowest in sepsis patients not surviving the disease. Also, other studies reported that deficiencies in specific amino acids were associated with mortality and other adverse outcomes in polymorbid patients at nutritional risk, suggesting that they may serve as potential prognostic biomarkers [12,13,15]. Nevertheless, different cancer studies also reported that high levels of methionine were associated with worse clinical outcomes and cancer growth. Ming et al. reported the inhibition of cancer growth and antitumor immunity when using a methionine-restrictive diet [41]. However, the underlying mechanism might differ from the population of malnourished polymorbid patients, since certain cancer cells have an increased need for methionine, and consequently, methionine restriction might be beneficial in that case [42]. Yet, interventional research is needed to further understand these effects. The role of methionine as a precursor of homocysteine, and the potentially increased risk of cardiovascular disease with elevated homocysteine is the subject of ongoing debate [43]. While an unrealistic, very high dosage of 1 g/kg body weight can result in death, a dosage five times higher than normal intake does elevate homocysteine. However, long-term data have indicated no adverse consequences of moderate fluctuations in dietary methionine intake, and the effects of methionine on homocysteine and vascular function are moderated by supplements of vitamins B-6, B-12, C, and folic acid [44].

Third, while lysine and threonine were not significantly associated with mortality, patients with low threonine levels had an increased risk of low skeletal muscle index, which is a surrogate for sarcopenia and has been shown to be an independent prognostic marker for worse clinical outcome, mainly in cancer patients [45,46]. Interestingly, low threonine was also associated with falls within 180 days. These findings may indicate a predominant role of threonine in muscle health in medical inpatients at nutritional risk, next to the anabolic effect of all essential amino acids via mTOR [47]. The role of threonine in muscle health has been investigated historically in the treatment of spasticity, with supplementation improving spasticity-related outcomes [48,49]. Mechanisms to explain this relationship are lacking, but cell and animal studies suggest that serine/threonine kinase 25 might play a key role [50,51].

Fourth, low lysine levels were associated with a lower probability of being sarcopenic, which is in contrast to what we have expected and available evidence since low levels of lysine were associated with an increased risk of sarcopenia [4,52]. This could be due to the fact that our study included a limited number of CT scans available, making the results more susceptible to statistical errors. As a limitation, we derived cut-off values from an oncological cohort displaying higher mean skeletal muscle mass and younger age may have led to the over-classification of sarcopenic patients in our cohort [36].

Finally, within this secondary analysis of a randomized trial, we also examined how patients with different levels of lysine, methionine, or threonine responded to nutritional interventions. We found no significant difference in the effectiveness of nutritional support between high and low admission concentrations of these amino acids. For all the investigated amino acids, however, patients with high levels appeared to respond better, indicated by a lower mortality rate through nutritional therapy in this subgroup. This is in line with previous secondary analyses, which showed that the positive effect of nutritional support diminishes with the increasing severity and acuity of a patient’s condition [11,15,53]. Consistently, the results from ICU studies did not show a positive effect of nutritional interventions in patients with high severity of illness [54,55]. This is also reflected in the current guidelines for polymorbid medical patients, which recommend using the severity of the acute phase to select patients for nutritional interventions [8]. Importantly, we did not use a diet specifically providing these amino acids in higher concentrations. Such interventional research is needed to understand whether supplementation may have positive clinical effects.

### Strengths and Limitations

The dataset used in this study originates from a randomized controlled trial, with a very well-characterized patient cohort with short- and long-term outcome measures in a prospective manner and follow-ups of over 5 years. It is the first analysis investigating lysine, methionine, and threonine by analyzing their influence on clinical outcomes and response to nutritional support in patients at nutritional risk. Since this is a secondary analysis, the results should be viewed as explorative and hypothesis-generating and require larger samples for confirmation. The analysis lacks external validity and power due to the monocentric approach and limitation to one subgroup within the center. Due to the small sample size regarding the muscle-specific variables, the 95% confidence intervals resulted in wide spans, representing lower precision. The implemented metabolomics measurement kit lacks well-validated reference values due to its application for research purposes only. Remaining confounding is plausible as a larger dataset would be needed, even though the analysis includes adjustment for confounding.

## 5. Conclusions

This secondary analysis of the randomized EFFORT suggests that methionine provides prognostic information on mortality in polymorbid medical inpatients at nutritional risk, while threonine showed associations with muscle health-specific outcomes. Since amino acid levels were not depleted only due to malnutrition, the underlying disease- and nutrition-specific mechanisms need to be further elucidated, to derive more targeted intervention in the future. This research contributes to the growing body of evidence supporting the critical role of personalized nutrition in patient care and recovery.

## Figures and Tables

**Table 1:** Baseline characteristics and stratified by 30-day all-cause mortality.

	Overall	No 30-Day Mortality	30-Day Mortality	*p*-Value
	n = 237	n = 179	n = 58	
Sociodemographic				
Male sex, n (%)	136 (57.4%)	94 (52.5%)	42 (72.4%)	0.008
Age, years, mean (SD)	73.4 (13.6)	72.5 (13.9)	76.1 (12.4)	0.083
Nutritional assessment, mean (SD)				
BMI, kg/m^2^	24.3 (5.0)	24.4 (5.2)	24.0 (4.0)	0.60
Weight, kg	68.7 (14.9)	68.7 (15.4)	68.8 (13.7)	0.96
Height, cm	168.1 (8.6)	167.8 (8.4)	169.1 (9.2)	0.33
Admission diagnosis, n (%)				
Infection	64 (27.0%)	50 (27.9%)	14 (24.1%)	0.57
Cancer	75 (31.6%)	50 (27.9%)	25 (43.1%)	0.031
Cardiovascular disease	24 (10.1%)	17 (9.5%)	7 (12.1%)	0.57
Frailty	13 (5.5%)	10 (5.6%)	3 (5.2%)	0.90
Lung disease	11 (4.6%)	6 (3.4%)	5 (8.6%)	0.097
Gastrointestinal disease	13 (5.5%)	13 (7.3%)	0 (0.0%)	0.035
Neurological disease	4 (1.7%)	4 (2.2%)	0 (0.0%)	0.25
Renal disease	15 (6.3%)	14 (7.8%)	1 (1.7%)	0.097
Metabolic disease	6 (2.5%)	5 (2.8%)	1 (1.7%)	0.65
Other	3 (1.3%)	2 (1.1%)	1 (1.7%)	0.72
Comorbidities, n (%)				
Hypertension	138 (58.2%)	107 (59.8%)	31 (53.4%)	0.40
Malignant disease	113 (47.7%)	78 (43.6%)	35 (60.3%)	0.026
Chronic kidney disease	81 (34.2%)	63 (35.2%)	18 (31.0%)	0.56
Coronary heart disease	54 (22.8%)	41 (22.9%)	13 (22.4%)	0.94
Diabetes	43 (18.1%)	33 (18.4%)	10 (17.2%)	0.84
Congestive heart failure	45 (19.0%)	30 (16.8%)	15 (25.9%)	0.12
Chronic obstructive pulmonary disease	28 (11.8%)	17 (9.5%)	11 (19.0%)	0.052
Peripheral arterial disease	26 (11.0%)	19 (10.6%)	7 (12.1%)	0.76
Cerebrovascular disease	27 (11.4%)	21 (11.7%)	6 (10.3%)	0.77
Dementia	11 (4.6%)	7 (3.9%)	4 (6.9%)	0.35
Amino acids				
Low lysine, n (%)	122 (51.5%)	97 (54.2%)	25 (43.1%)	0.14
Low methionine, n (%)	70 (29.5%)	46 (25.7%)	24 (41.4%)	0.023
Low threonine, n (%)	110 (46.4%)	86 (48.0%)	24 (41.4%)	0.38

SD, standard deviation. NRS, Nutritional Risk Screening 2002.

**Table 2:** Association of nutritional parameters with admission levels of lysine, methionine, and threonine.

	Lysine				Methionine				Threonine			
	Mean	Coef.	*p*-Value	95%-CI	Mean	Coef.	*p*-Value	95%-CI	Mean	Coef.	*p*-Value	95%-CI
NRS 2002 score												
3	211.1	reference			25.3	reference			103.0	reference		
4	199.1	−12.0	0.23	−31.4 to 7.4	22.5	−2.8	0.08	−6.0 to 0.4	96.8	−6.2	0.34	−19.0 to 6.6
≥5	186.7	−24.4	0.01	−43.0 to −5.8	22.6	−2.7	0.09	−5.7 to 0.4	96.4	−6.6	0.29	−18.9 to 5.7
Food intake												
>75%	215.9	reference			25.8	reference			104.2	reference		
50–75%	185.7	−30.2	0.03	−58.0 to −2.4	22.9	−2.9	0.22	−7.4 to 1.7	94.8	−9.5	0.31	−27.8 to 8.9
25–50%	202.0	−13.9	0.30	−40.4 to 12.6	22.8	−2.9	0.19	−7.2 to 1.4	97.2	−7.1	0.43	−24.5 to 10.4
0–25%	193.7	−22.2	0.14	−51.6 to 7.1	23.4	−2.3	0.34	−7.1 to 2.5	102.7	−1.5	0.88	−20.8 to 17.8
BMI												
<18.5	178.7	reference			23.2	reference			91.4	reference		
18.5–25	200.1	21.4	0.11	−4.9 to 47.7	22.9	−0.3	0.88	−4.7 to 4.0	98.2	6.8	0.44	−10.4 to 24.1
25–30	195.2	16.5	0.25	−11.8 to 44.9	22.9	−0.3	0.90	−4.9 to 4.4	95.2	3.8	0.69	−14.8 to 22.5
>30	205.0	26.3	0.09	−3.7 to 56.2	24.6	1.4	0.58	−3.5 to 6.3	106.9	15.5	0.12	−4.2 to 35.2
Weight loss												
1 (none)	201.0	reference			23.1	reference			97.6	reference		
2 (>5% in 3 mts)	197.4	−3.6	0.74	−24.4 to 17.3	23.8	0.7	0.70	−2.7 to 4.1	100.6	3.0	0.67	−10.7 to 16.6
3 (>5% in 2 mts)	201.7	0.7	0.95	−20.9 to 22.3	24.9	1.8	0.31	−1.7 to 5.3	104.5	6.9	0.34	−7.3 to 21.0
4 (>5% in 1 mts)	184.6	−16.4	0.11	−36.6 to 3.8	21.7	−1.5	0.38	−4.8 to 1.8	92.4	−5.2	0.44	−18.5 to 8.0
Disease severity												
0	182.0	reference			27.2	reference			132.0	reference		
1	198.4	16.4	0.63	−51.1 to 83.9	23.4	−3.8	0.49	−14.8 to 7.1	99.3	−32.7	0.14	−76.7 to 11.3
2	194.6	12.6	0.72	−55.5 to 80.6	23.0	−4.2	0.45	−15.3 to 6.8	95.3	−36.7	0.10	−81.1 to 7.7
3	274.0	92.0	0.18	−41.6 to 225.6	14.0	−13.2	0.23	−34.9 to 8.5	84.3	−47.7	0.28	−134.8 to 39.5

NRS, Nutritional Risk Screening 2002. Mts, months.

**Table 3:** Prognostic value of low lysine, methionine, and threonine to predict clinical outcomes.

**All-Cause Mortality**		**Low Plasma Levels**	**High Plasma Levels**	**HR (95% CI)**	***p*-Value**
30-day mortality					
	Lysine	25/122 (20.5%)	33/115 (28.7%)	0.69 (0.40–1.18)	0.17
	Methionine	24/70 (34.3%)	34/167 (20.4%)	1.98 (1.16–3.36)	0.01
	Threonine	24/110 (21.8%)	34/127 (26.8%)	0.77 (0.45–1.30)	0.33
180-day mortality					
	Lysine	54/122 (44.3%)	48/115 (41.7%)	1.02 (0.68–1.53)	0.93
	Methionine	35/70 (50.0%)	67/167 (40.1%)	1.45 (0.95–2.22)	0.08
	Threonine	48/110 (43.6%)	54/127 (42.5%)	0.85 (0.57–1.27)	0.43
**Other Clinical Outcomes**		**Low Plasma Levels**	**High Plasma Levels**	**OR (95% CI)**	***p*-Value**
Adverse event 30 days					
	Lysine	44/122 (36.0%)	48/115 (41.7%)	0.72 (0.41–1.27)	0.26
	Methionine	36/70 (51.4%)	56/167 (33.5%)	2.21 (1.21–4.05)	0.01
	Threonine	42/110 (38.2%)	50/127 (39.4%)	0.86(0.09–8.49)	0.60
Barthel Decline > 10					
	Lysine	31/122 (25.4%)	34/115 (29.6%)	0.77 (0.41–1.46)	0.42
	Methionine	26/70 (37.1%)	39/167 (23.6%)	2.06 (1.06–4.01)	0.03
	Threonine	29/110 (26.4%)	36/127 (28.4%)	0.79 (0.42–1.48)	0.46
Falls 180 days					
	Lysine	14/121 (11.6%)	9/115 (7.8%)	1.47 (0.59–3.62)	0.41
	Methionine	8/70 (11.4%)	15/166 (9.0%)	1.26 (0.50–3.20)	0.62
	Threonine	15/109 (13.8%)	8/127 (6.3%)	2.46 (0.98–6.19)	0.05
**Muscle-Specific Outcomes**		**Low Plasma Levels**	**High Plasma Levels**	**OR (95% CI)**	***p*-Value**
Sarcopenia					
	Lysine	17/72 (23.6%)	22/81 (27.2%)	0.37 (0.14–0.95)	0.04
	Methionine	12/43 (27.9%)	27/110 (24.5%)	0.53 (0.19–1.47)	0.22
	Threonine	23/78 (29.5%)	16/75 (21.3%)	0.96 (0.39–2.35)	0.93
Low skeletal muscle index					
	Lysine	26/33 (78.8%)	20/28 (71.4%)	1.46 (0.40–5.27)	0.57
	Methionine	15/18 (83.3%)	31/43 (72.1%)	2.05 (0.46–9.08)	0.35
	Threonine	28/33 (84.8%)	18/28 (64.3%)	3.14 (0.89–11.02)	0.07
Low muscle radiodensity					
	Lysine	27/33 (81.8%)	15/28 (53.6%)	2.48 (0.63–9.85)	0.20
	Methionine	15/18 (83.3%)	27/43 (62.8%)	2.88 (0.48–17.29)	0.25
	Threonine	26/33 (78.8%)	16/28 (57.1%)	2.93 (0.72–12.00)	0.13
High intramuscular adipose tissue					
	Lysine	11/33 (33.3%)	4/28 (14.3%)	2.37 (0.14–10.06)	0.24
	Methionine	4/18 (22.2%)	11/43 (25.5%)	0.54 (0.12–2.34)	0.41
	Threonine	8/33 (24.2%)	7/28 (25.0%)	0.69 (0.19–2.54)	0.58

HRs (hazard ratios) and ORs (odds ratios) were adjusted for CCI, sex, NRS total score, and intervention. CCI, Charlson Comorbidity Index; NRS, Nutritional Risk Screening 2002.

## Data Availability

Our data will be made available to others with the publication of this manuscript, as already outlined in the primary EFFORT publication, on receipt of a letter of intention detailing the study hypothesis and statistical analysis plan. A signed data access agreement is required from all applicants. Please send requests to the principal investigator of this trial.

## Supplementary Materials

The following supporting information can be downloaded at: https://www.mdpi.com/article/10.3390/nu16162608/s1, Figure S1: Study flow chart of the secondary analysis based on Schuetz et al., 2019; Table S1: Baseline characteristics lysine; Table S2: Baseline characteristics methionine; Table S3: Baseline characteristics threonine; Figure S2: 30-day mortality Kaplan-Meier curves for (A) lysine, (B) threonine.

## Abbreviations

BMI, body mass index; CCI, Charlson Comorbidity Index; CI, confidence interval; CT, computer tomography; DRM, disease-related malnutrition; EAA, essential amino acid; EFFORT, Effect of Early Nutritional Support on Frailty, Functional Outcomes, and Recovery of Malnourished Medical Inpatients Trial; GLIM, Global Leadership Initiative on Malnutrition; HGS, hand grip strength; HR, hazard ratio; HU, Hounsfield unit; ICU, intensive care unit; IMAT, intramuscular adipose tissue; MR, muscle radiodensity; mTOR, mammalian target of rapamycin; NRS, Nutritional Risk Screening; OR, odds ratio; RCT, randomized clinical trial; SD, standard deviation; SMI, skeletal muscle index.
