# Supplementary material for: The Association of the Essential Amino Acids Lysine, Methionine, and Threonine with Clinical Outcomes in Patients at Nutritional Risk: Secondary Analysis of a Randomized Clinical Trial
Source: Nutrients. 2024 Aug 8;16(16):2608. doi: 10.3390/nu16162608 (PMC11357570; doi:10.3390/nu16162608)
Supplement: Supplementary file 1 [file nutrients-16-02608-s001.zip › Supplemental Files.pdf]

Figure S1: Study flow chart of the secondary analysis based on Schuetz et al., 2019

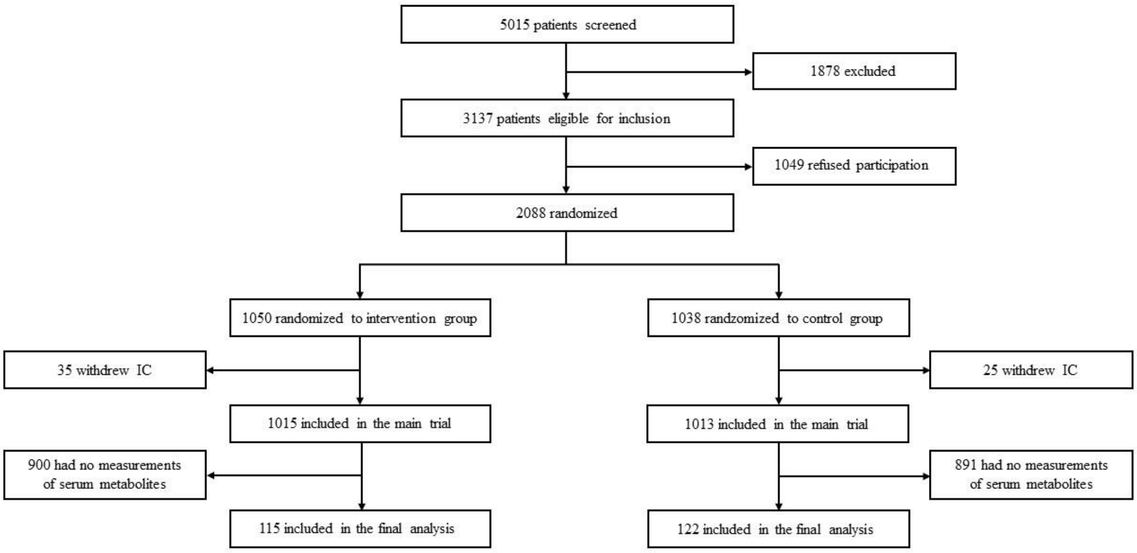

Figure S1: Study flow chart of the secondary analysis based on Schuetz et al., 2019 [9]; IC, informed consent. A total of 237 available metabolite measurements. Reasons for exclusion: 145 surgical patients, 268 unable to ingest oral nutrition, 158 with terminal condition, 719 already receiving nutritional therapy, 31 anorexia nervosa, 161 acute pancreatitis, 81 acute liver failure, 6 cystic fibrosis, 11 stem-cell transplantation, 27 post gastric bypass operation, 43 contraindications against nutritional support, 228 earlier inclusions.

Table S1: Baseline characteristics lysine

	Overall<br>n = 237	High lysine > 192.5 µmol/L<br>n = 115	Low lysine ≤ 192.5 µmol/L<br>n = 122	p-value
**Sociodemographics**				
Male sex, n (%)	136 (57.4%)	71 (61.7%)	65 (53.3%)	0.19
Age, years, mean (SD)	73.4 (13.6)	73.3 (13.2)	73.5 (14.0)	0.88
**Nutritionnal assessment, mean (SD)**				
BMI, kg/m^2	24 (5)	25 (5)	24 (5)	**0.019**
Weight, kg	69 (15)	71 (15)	67 (14)	**0.023**
Height, cm	168.1 (8.6)	168.3 (8.7)	167.9 (8.5)	0.68
**NRS 2002 score, n (%)**				0.30
3 points	62 (26.2%)	34 (29.6%)	28 (23.0%)	
4 points	79 (33.3%)	40 (34.8%)	39 (32.0%)	
≥ 5 points	96 (40.5%)	41 (35.7%)	55 (45.1%)	
**Musclefunction, mean (SD)**				
Handgrip strength, kg	23.7 (11.6)	23.8 (12.2)	23.6 (11.0)	0.91
**Admission diagnosis, n (%)**				
Infection	64 (27.0%)	31 (27.0%)	33 (27.0%)	0.99
Cancer	75 (31.6%)	30 (26.1%)	45 (36.9%)	0.074
Cardiovascular disease	24 (10.1%)	16 (13.9%)	8 (6.6%)	0.061
Frailty	13 (5.5%)	6 (5.2%)	7 (5.7%)	0.86
Lung disease	11 (4.6%)	8 (7.0%)	3 (2.5%)	0.10
Gastrointestinal disease	13 (5.5%)	8 (7.0%)	5 (4.1%)	0.33
Neurological disease	4 (1.7%)	1 (0.9%)	3 (2.5%)	0.34
Renal disease	15 (6.3%)	8 (7.0%)	7 (5.7%)	0.70
Metabolic disease	6 (2.5%)	3 (2.6%)	3 (2.5%)	0.94
Other	3 (1.3%)	2 (1.7%)	1 (0.8%)	0.53
**Comorbidities, n (%)**				
Hypertension	138 (58.2%)	71 (61.7%)	67 (54.9%)	0.29
Malignant disease	113 (47.7%)	51 (44.3%)	62 (50.8%)	0.32
Chronic kidney disease	81 (34.2%)	43 (37.4%)	38 (31.1%)	0.31
Coronary heart disease	54 (22.8%)	28 (24.3%)	26 (21.3%)	0.58
Diabetes	43 (18.1%)	27 (23.5%)	16 (13.1%)	**0.039**
Congestive heart failure	45 (19.0%)	22 (19.1%)	23 (18.9%)	0.96
Chronic obstructive pulmonary disease	28 (11.8%)	16 (13.9%)	12 (9.8%)	0.33
Peripheral arterial disease	26 (11.0%)	12 (10.4%)	14 (11.5%)	0.80
Cerebrovascular disease	27 (11.4%)	12 (10.4%)	15 (12.3%)	0.65
Dementia	11 (4.6%)	6 (5.2%)	5 (4.1%)	0.68

Table S1: Baseline characteristics stratified by lysine at cut-off value 192.5 µmol/L. SD, standard deviation. NRS, Nutritional Risk Screening 2002.

Table S2: Baseline characteristics methionine

	Overall	High methionine > 17.45 µmol/L	Low methionine ≤ 17.45 µmol/L	p-value
	n = 237	n = 167	n= 70	
**Sociodemographics**				
Male sex, n (%)	136 (57.4%)	100 (59.9%)	36 (51.4%)	0.23
Age, years, mean (SD)	73.4 (13.6)	72.4 (13.7)	75.7 (13.0)	0.086
**Nutritionnal assessment, mean (SD)**				
BMI, kg/m^2	24 (5)	24 (5)	24 (5)	0.41
Weight, kg	69 (15)	69 (15)	67 (14)	0.31
Height, cm	168.1 (8.6)	168.3 (8.7)	167.7 (8.4)	0.67
**NRS 2002 score, n (%)**				0.56
3 points	62 (26.2%)	47 (28.1%)	15 (21.4%)	
4 points	79 (33.3%)	54 (32.3%)	25 (35.7%)	
≥5 points	96 (40.5%)	66 (39.5%)	30 (42.9%)	
**Musclefunction, mean (SD)**				
Handgrip strength, kg	23.7 (11.6)	24.0 (11.3)	22.9 (12.3)	0.60
**Admission diagnosis, n (%)**				
Infection	64 (27.0%)	43 (25.7%)	21 (30.0%)	0.50
Cancer	75 (31.6%)	54 (32.3%)	21 (30.0%)	0.72
Cardiovascular disease	24 (10.1%)	19 (11.4%)	5 (7.1%)	0.32
Frailty	13 (5.5%)	9 (5.4%)	4 (5.7%)	0.92
Lung disease	11 (4.6%)	7 (4.2%)	4 (5.7%)	0.61
Gastrointestinal disease	13 (5.5%)	11 (6.6%)	2 (2.9%)	0.25
Neurological disease	4 (1.7%)	3 (1.8%)	1 (1.4%)	0.84
Renal disease	15 (6.3%)	8 (4.8%)	7 (10.0%)	0.13
Metabolic disease	6 (2.5%)	5 (3.0%)	1 (1.4%)	0.48
Other	3 (1.3%)	2 (1.2%)	1 (1.4%)	0.88
**Comorbidities, n (%)**				
Hypertension	138 (58.2%)	96 (57.5%)	42 (60.0%)	0.72
Malignant disease	113 (47.7%)	79 (47.3%)	34 (48.6%)	0.86
Chronic kidney disease	81 (34.2%)	60 (35.9%)	21 (30.0%)	0.38
Coronary heart disease	54 (22.8%)	41 (24.6%)	13 (18.6%)	0.32
Diabetes	43 (18.1%)	34 (20.4%)	9 (12.9%)	0.17
Congestive heart failure	45 (19.0%)	31 (18.6%)	14 (20.0%)	0.80
Chronic obstructive pulmonary disease	28 (11.8%)	19 (11.4%)	9 (12.9%)	0.75
Peripheral arterial disease	26 (11.0%)	18 (10.8%)	8 (11.4%)	0.88
Cerebrovascular disease	27 (11.4%)	17 (10.2%)	10 (14.3%)	0.36
Dementia	11 (4.6%)	8 (4.8%)	3 (4.3%)	0.87

Table S2: Baseline characteristics stratified by methionine at cut-off value 17.45 µmol/L. SD, standard deviation. NRS, Nutritional Risk Screening 2002.

Table S3: Baseline characteristics threonine

	Overall<br>n=237	High threonine > 88.15 μmol/L<br>n=127	Low threonine ≤ 88.15 μmol/L<br>n=110	p-value
**Sociodemographics**				
Male sex, n (%)	136 (57.4%)	76 (59.8%)	60 (54.5%)	0.41
Age, years, mean (SD)	73.4 (13.6)	73.3 (13.2)	73.6 (14.0)	0.87
**Nutritionnal assessment, mean (SD)**				
BMI, kg/m^2	24 (5)	25 (5)	24 (5)	0.11
Weight, kg	69 (15)	70 (15)	67 (14)	0.095
Height, cm	168.1 (8.6)	168.3 (8.6)	167.9 (8.7)	0.67
**NRS 2002 score, n (%)**				0.37
3 points	62 (26.2%)	38 (29.9%)	24 (21.8%)	
4 points	79 (33.3%)	40 (31.5%)	39 (35.5%)	
≥5 points	96 (40.5%)	49 (38.6%)	47 (42.7%)	
**Musclefunction, mean (SD)**				
Handgrip strength, kg	23.7 (11.6)	23.5 (9.7)	23.8 (13.3)	0.90
**Admission diagnosis, n (%)**				
Infection	64 (27.0%)	33 (26.0%)	31 (28.2%)	0.70
Cancer	75 (31.6%)	35 (27.6%)	40 (36.4%)	0.15
Cardiovascular disease	24 (10.1%)	14 (11.0%)	10 (9.1%)	0.62
Frailty	13 (5.5%)	9 (7.1%)	4 (3.6%)	0.24
Lung disease	11 (4.6%)	8 (6.3%)	3 (2.7%)	0.19
Gastrointestinal disease	13 (5.5%)	7 (5.5%)	6 (5.5%)	0.98
Neurological disease	4 (1.7%)	3 (2.4%)	1 (0.9%)	0.39
Renal disease	15 (6.3%)	8 (6.3%)	7 (6.4%)	0.98
Metabolic disease	6 (2.5%)	3 (2.4%)	3 (2.7%)	0.86
Other	3 (1.3%)	2 (1.6%)	1 (0.9%)	0.65
**Comorbidities, n (%)**				
Hypertension	138 (58.2%)	74 (58.3%)	64 (58.2%)	0.99
Malignant disease	113 (47.7%)	56 (44.1%)	57 (51.8%)	0.24
Chronic kidney disease	81 (34.2%)	44 (34.6%)	37 (33.6%)	0.87
Coronary heart disease	54 (22.8%)	29 (22.8%)	25 (22.7%)	0.98
Diabetes	43 (18.1%)	22 (17.3%)	21 (19.1%)	0.72
Congestive heart failure	45 (19.0%)	22 (17.3%)	23 (20.9%)	0.48
Chronic obstructive pulmonary disease	28 (11.8%)	16 (12.6%)	12 (10.9%)	0.69
Peripheral arterial disease	26 (11.0%)	14 (11.0%)	12 (10.9%)	0.98
Cerebrovascular disease	27 (11.4%)	15 (11.8%)	12 (10.9%)	0.83
Dementia	11 (4.6%)	6 (4.7%)	5 (4.5%)	0.95

Table S3: Baseline characteristics stratified by threonine at cut-off value 88.15 μmol/L. SD, standard deviation. NRS, Nutritional Risk Screening 2002.

Figure S2: 30-day mortality Kaplan-Meier curves for A) lysine, B) threonine

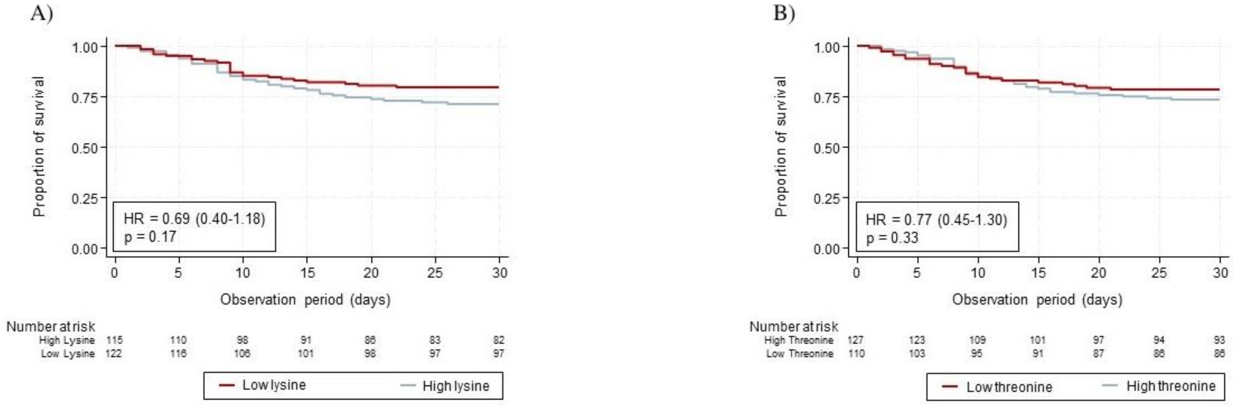

Figure S2: Kaplan-Meier estimate for 30-day all-cause mortality according to cut off values A) lysine 192.5 μmol/L, B) threonine 88.15 μmol/L. HR, hazard ratio. All HR shown are adjusted for CCI, sex, NRS total score, and intervention.
